# Comprehensive characterization of MUC16 mutations in lung adenocarcinoma for immunotherapies and prognosis: An observational study

**DOI:** 10.1097/MD.0000000000035481

**Published:** 2023-11-03

**Authors:** Tingjun Liu, Lianlian Wu, Jing Liu, Hao Chen, Bao Zhu, Dandan Qiao, Yuhua Zhu, Tingya Liu, Quangang Chen, Ankang Hu

**Affiliations:** a Center of Animal Laboratory, Xuzhou Medical University, Xuzhou, Jiangsu, China; b Department of Respiratory Medicine, Xuzhou Central Hospital, Xuzhou, Jiangsu, China; c Department of Respiratory Medicine, The Affiliated Hospital of Xuzhou Medical University, Xuzhou, China; d Cancer Institute, Xuzhou Medical University, Xuzhou, Jiangsu Province, Xuzhou, China; e Department of Neurology, The Affiliated Hospital of Xuzhou Medical University, Xuzhou, China; f School of Life Sciences, Xuzhou Medical University, Xuzhou, Jiangsu, China.

**Keywords:** immune prognostic model, lung adenocarcinoma, MUC16, tumor infiltrating immune cells, tumor mutation burden

## Abstract

Lung adenocarcinoma (LUAD) is a non-small-cell lung cancer and is the leading cause of cancer-related deaths worldwide. Immunotherapy is a promising candidate for LUAD, and tumor mutation burden (TMB) could be a new biomarker to monitor the response of cancer patients to immunotherapy. It is known that the mucin 16 (MUC16) mutation is the most common and affects the progression and prognosis of several cancers. However, whether MUC16 mutations are associated with TMB and tumor-infiltrating immune cells in LUAD is not fully elucidated. All the data were obtained from the cancer genome atlas database to assess the prognostic value and potential mechanism of MUC16 in LUAD. An immune prognostic model (IPM) was developed based on immune-related genes that could be differentially expressed between MUC16^MUT^ and MUC16^WT^ LUAD patients. Later, the IPM effect on the prognosis and immunotherapy of LUAD was comprehensively evaluated. MUC16 was frequently mutated in LUAD, with a mutational frequency of 43.4%, significantly associated with higher TMB and better clinical prognosis. Based on 436 patients with LUAD, an IPM was established and validated to differentiate patients with a low or high risk of poor survival. The univariate and multivariate Cox regression analyses demonstrated that the IPM was an independent prognostic indicator for LUAD patients. Elevated expressions of PD-L1, LAG3, PDCD1, and SIGLEC15, and most of the T-effector and interferon-γ gene signatures, were depicted in the high-risk group. Moreover, the nomogram using the IPM and clinical prognostic factors also predicted the overall survival and clinical utility. Our project developed a robust risk signature depending on the MUC16 status and provided novel insights for individualized treatment options for LUAD patients.

## 1. Introduction

Lung cancer ranks among the top ten causes of cancer-related deaths in both men and women. Lung adenocarcinoma (LUAD) is makes up approximately half of all lung cancers.^[1,2]^ Even though cancer immunotherapy provides new treatment options by integrating conventional and targeted therapies, such as immune checkpoint inhibitors (ICIs), which can block the inhibitory programmed cell death protein 1 and programmed death ligand 1 (PD-1/PD-L1) immune checkpoint axis and has a prominent and durable response in some LUAD patients.^[3]^ However only a subset of LUAD patients could benefit from ICI treatment.^[4]^ This is due to that more than one-half of patients are either insensitive or relapse after a response period, seriously limiting ICI effectiveness.^[5]^ Therefore, identifying the predictive biomarkers for ICI response can help explore strategies for tumor immunotherapy.

Tumor mutation burden (TMB) depicts the total number of somatic mutations on a cancer genome per megabase. A non-synonymous somatic mutation can contribute to cancer development and cause the immune system to mount an antitumor response to the tumor.^[6]^ Recent studies have depicted that TMB was significantly positively associated with immune checkpoint blockade across 27 cancer types.^[7]^ Identifying the driver mutations in the tumor cells of a cancer patient is crucial in precision cancer treatment.^[8]^

Several genetic variants have been reported to affect the relative risk of different cancers. This includes the CUB and Sushi multiple domains protein 3 (CSMD3) mutations in ovarian cancer,^[9]^ the mucin 4 mutations in colon cancer,^[10]^ ryanodine receptor 2 in breast cancer,^[11]^ tumor protein P53 (TP53) in prostate cancer and hepatocellular carcinoma,^[12]^ etc. Furthermore, titin (TTN),^[13]^ TP53, KRAS proto-oncogene and GTPase (KRAS),^[14]^ epidermal growth factor receptor,^[15]^ Kelch-like ECH-associated protein (KEAP1)^[16]^ have significant relevance with the carcinogenesis and prognosis in LUAD patients.

Carcinoma antigen-125 (CA125), also known as mucin 16 (MUC16), is a glycoprotein from the mucin family. It is found on the surface of many ovarian cancer cells.^[17]^ MUC16 expression correlates with disease progression and metastasis, such as pancreatic cancer, colorectal cancer, and gastric adenocarcinoma.^[18,19]^ Recent studies have revealed that MUC16 is one of the most frequently mutated genes in hepatocellular carcinoma,^[20]^ gastric cancers,^[21]^ and melanoma.^[22]^ However, no studies have described the MUC16 mutations and their association with TMB and tumor-infiltrating immune cells in LUAD patients.

The present study aims to explore the association of MUC16 mutations with TMB and prognosis in LUAD patients. The results indicate that MUC16 mutations are closely linked to LUAD patients and can act as biomarkers to forecast immune response.

## 2. Materials and methods

### 2.1. Data acquisition

We downloaded the normalized RNA-sequencing dataset (N = 502), somatic mutation (N = 508), and the associated clinical information of the LUAD samples (N = 522) from the cancer genome atlas (TCGA) databases (https://portal.gdc.cancer.gov/). From these data, 508 samples with RNA-sequencing data and MUC16 mutation information were subjected to subsequent analyses. We retrieved 462 patients from the Gene Expression Omnibus for the validation set (GEO; accession number: GSE68465; https://www.ncbi.nlm.nih.gov/geo/query/acc.cgi?acc=GSE68465).

### 2.2. TMB calculation in LUAD patients

TMB depicts the number of somatic, coding, substitution, and indel mutations per megabase of the examined genome. The total number of somatic mutations was divided by the size of the TMB scores depending on the exome size.^[23]^

### 2.3. Gene set enrichment analysis (GSEA)

GSEA is used to determine whether a particular gene set differs significantly between the LUAD samples with (n = 201) and without (n = 307) MUC16 mutations in the TCGA LUAD cohort during the MSigDB Collection enrichment (c5.go.bp.v7.4.symbols.gmt). The GSEA software (version 3.0) was obtained from its website (DOI:10.1073/pnas.0506580102, http://software.broadinstitute.org/gsea/index.jsp). The gene sets having a nominal *P* < .05 were statistically significant.

### 2.4. Identification of differentially expressed genes (DEGs) and functional enrichment analysis

We compared the 307 LUAD samples without and 201 LUAD samples with MU16 mutations to identify the DEGs with the edgeR R. package. The screening criteria for mRNAs differential expression were determined as the adjusted *P* < .05 and | fold change| >1.5.

Gene ontology (GO) and the Kyoto encyclopedia of genes and genomes (KEGG) analyses were conducted to compare the differential signaling pathways and biological effects among high-risk and low-risk groups. The enrichment analysis was performed with the R package clusterprofiler (version 3.14.3) to obtain the gene set enrichment results.

### 2.5. Construction and validation of the immune-related prognostic model

An immune-related prognostic model was constructed utilizing the regression coefficients derived from multivariate Cox regression analysis to multiply the expression level of each immune gene.^[24]^ X-tile 3.6.1 software (Yale University, New Haven, CT) was applied to determine the best cutoff for LUAD patients classified as low risk and high risk (it covers the MUC16 mutation). Additionally, the Kaplan–Meier survival analysis calculated the differences in overall survival (OS) between the high-risk and low-risk groups using a log-rank test in the “survival” R package. We calculated the receiver operating characteristic analysis (ROC) and the area under the ROC curve (AUC) through “version 1.17.0.1” of the R package to evaluate the predictive efficiency of the immune prognostic model (IPM).

### 2.6. Estimation of immune cell type fractions

The CIBERSORT algorithm can identify cellular biomarkers and novel therapeutic targets by discriminating the 22 human immune cell phenotypes.^[25]^ The matrix data visualization was performed using the R package “corrplot.” The landscape map demonstrated the difference in immune infiltration between the high-risk and low-risk groups for the 22 immune cell types.

### 2.7. Independence of the IPM from conventional clinicopathological factors

Among the 522 LUAD samples with survival information, 436 with complete clinical information, such as age, gender, MUC16 mutant status, TNM stage, survival data, and risk score, were subjected to subsequent analysis. The univariate and multivariate COX regression analyses investigated whether IPM was an independent prognostic factor among the other clinicopathological factors.^[26]^

### 2.8. Development and validation of the nomogram model

The R software package “rms” integrated the survival time data, survival status, and 7 features. Then, a nomogram was established using the cox method to assess the prognostic significance of these features among the 436 samples. The calibration curves were determined by mapping the predicted probabilities against the observed events, and the 45° line represented the most accurate prediction.^[27]^ The ROC curve with AUC value was generated using the “survival ROC” R package to evaluate the clinical utility of the nomogram.

### 2.9. Statistical analysis

The statistical analyses were conducted with R software version 4.0.2. Kaplan–Meier survival curves were analyzed using the log-rank test on the survival curves. Univariate and multivariate Cox regression analyses were used for identifying prognosis risk factors. The significance level was set at 0.05 in the 2-tailed statistical tests.

## 3. Results

### 3.1. The correlation between MUC16 mutations and immune phenotype in LUAD

**Figure 1.**
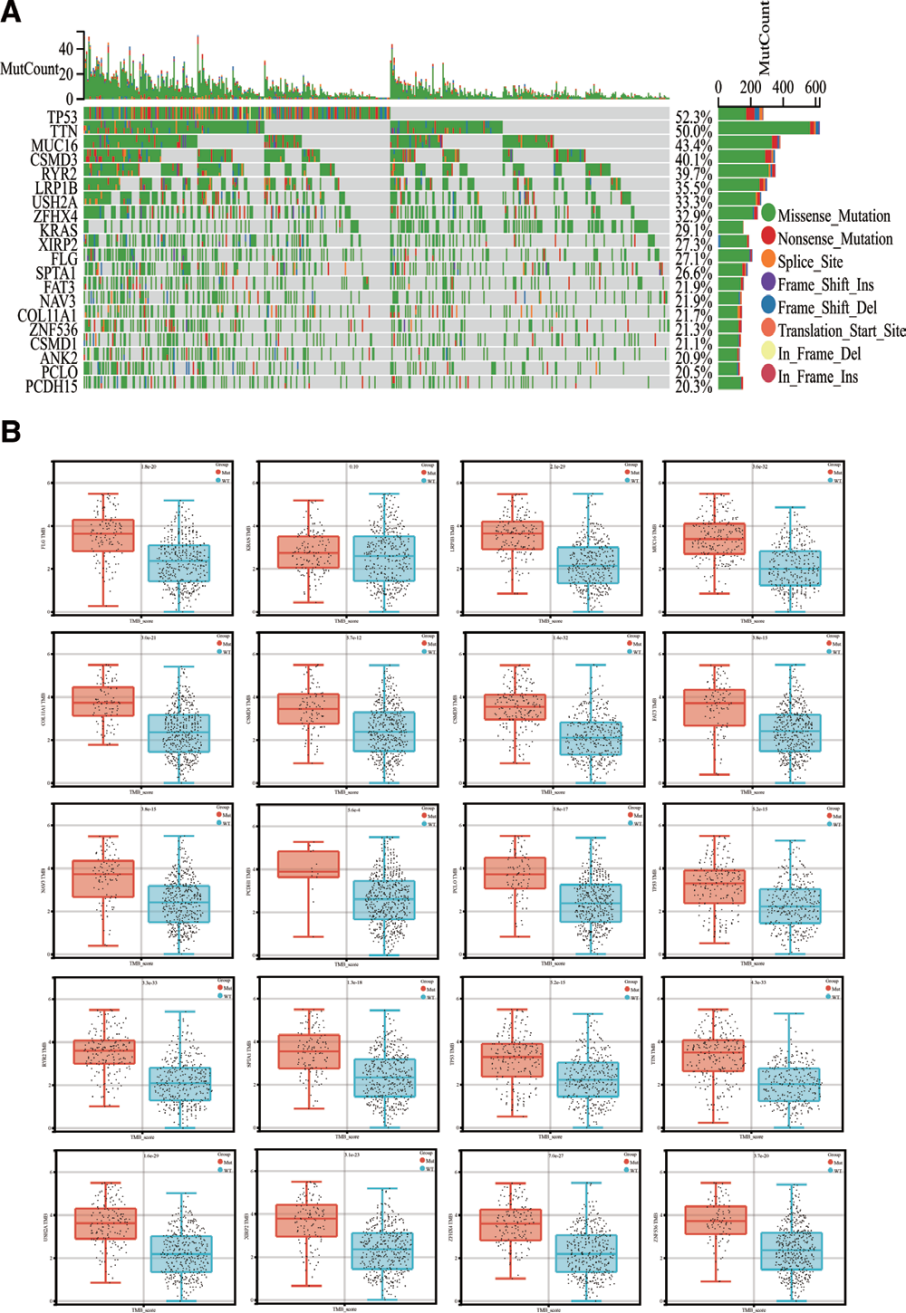
Overview of the frequently mutated genes in lung adenocarcinoma (LUAD). (A) The frequently mutated genes among the LUAD specimens obtained from the cancer genome atlas (TCGA) dataset are depicted in the waterfall plot. (B) The association between the gene mutations and tumor mutation burden (TMB).

Recognizing mutation characteristics is necessary for understanding how mutation functions during LUAD pathogenesis. As demonstrated in Figure 1A, the top 20 genes showing high mutation frequency in LUAD patients from the TCGA databases have been illustrated in waterfall plots. The TP53 mutant had the highest frequency (52.3%) among LUAD patients, followed by TTN (50.0%), MUC16 (43.4%), CSMD3 (40.1%), and ryanodine receptor 2 (39.7%). Mutations of these genes were missense mutations. LUAD patients from TCGA database were assigned to the wild-type or mutation groups based on the 20 gene mutation status to explore further the correlation between these highly mutated genes and TMB. It was observed that the TMB value in the mutation group of all the other 19 genes except KRAS had a significantly higher TMB than in the wild-type groups (Fig. 1B).

**Figure 2.**
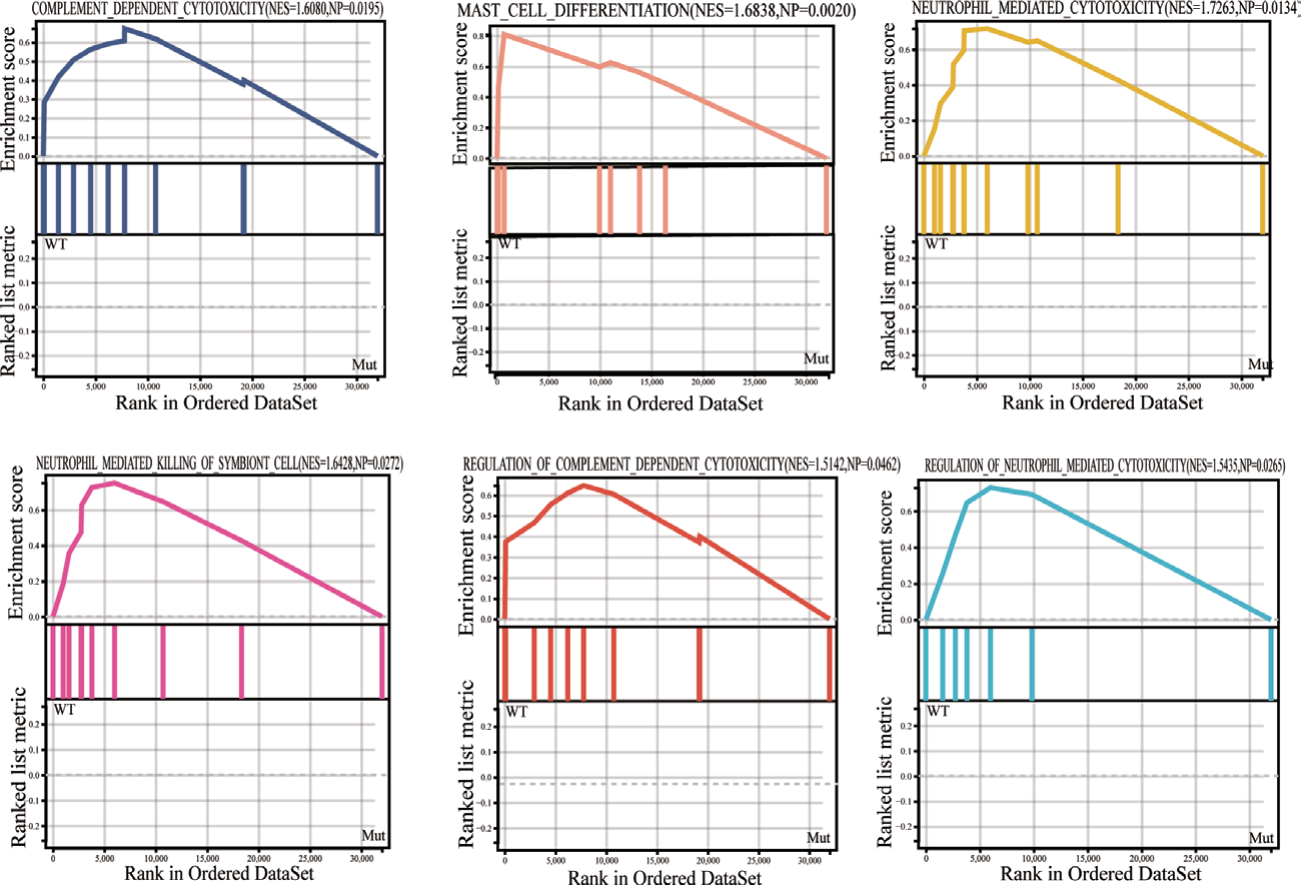
Gene set enrichment analysis (GSEA) enrichment analysis. Gene enrichment plots indicate that a series of immune-related gene sets are enriched within the MUC16^WT^ group. MUC16 = mucin 16, NES = normalized enrichment score, *P =* nominal *P* value.

The pathogenetic role of MUC16 mutations in the prognosis of patients having cutaneous melanoma, gastric cancer, hepatocellular carcinoma, and cervical cancer has been well reported. However, the relationship between MUC16 mutation and immune response has not been thoroughly examined. Therefore, according to gene expression data and clinical information extracted, GSEA was used to estimate the immune-associated biological processes between MUC16^MUT^ (n = 201) and MUC16^WT^ (n = 307) LUAD patients from the TCGA database. Based on the GSEA analysis, MUC16^WT^ LUAD patients indicated enrichment in 173 biological processes (see Table S1, Supplemental Content, http://links.lww.com/MD/K146 173 biological processes), 6 of which were immune-related, such as REGULATION_OF_COMPLEMENT_DEPENDENT_CYTOTOXICITY (normalized enrichment score, NES = 1.5142, *P* = .0462), REGULATION_OF_NEUTROPHIL_MEDIATED_CYTOTOXICITY (NES = 1.5435, *P* = .0265), NEUTROPHIL_MEDIATED_KILLING_OF_SYMBIONT_CELL (NES = 1.6428, *P* = .0272), COMPLEMENT_DEPENDENT_CYTOTOXICITY (NES = 1.608, *P* = .0195), MAST_CELL_DIFFERENTIATION (NES = 1.6838, *P* = .002), and NEUTROPHIL_MEDIATED_CYTOTOXICITY (NES = 1.7263, *P* = .0134) (Fig. 2).

### 3.2. The establishment of an IPM and assessment of its predictive ability within the TCGA LUAD cohort

We intended to evaluate the predictability of the DEGs due to the differences in immune status between MUC16^MUT^ and MUC16^WT^ LUADs. Simultaneously, there were 497 MUC16 status-associated DEGs, including 335 downregulated and 162 upregulated DEGs in

**Figure 3.**
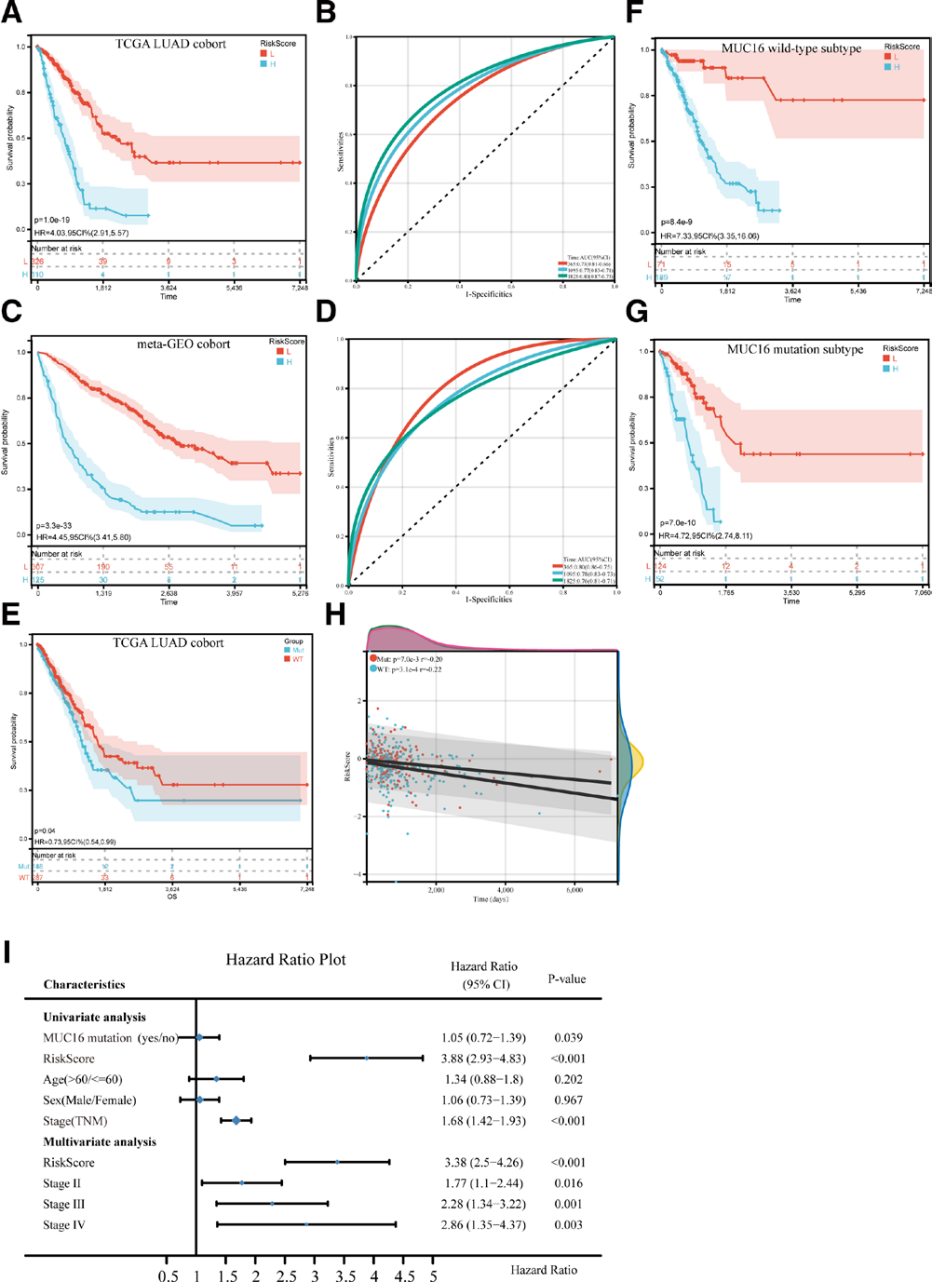
The prognostic analysis of immune prognostic model (IPM) and mucin 16 (MUC16) mutation. (A and B) Risk scores distribution, survival status of each patient, and the time-dependent receiver operating characteristic (ROC) curves of IPM for the cancer genome atlas (TCGA) lung adenocarcinoma (LUAD) and meta-GEO LUAD cohorts (C and D). (E–G) Kaplan–Meier survival of MUC16 status (E), the MUC16 mutation subgroup (F), and the MUC16 wild-type subgroup (G). (H) The correlation between risk score and survival time was analyzed based on the MUC16 status. (I) Univariate and multivariate analyses to assess the association between IPM and the conventional prognostic factors.

MUC16^MUT^ LUADs (*P* < .05 and |log2FC| > 1.5) (see Table S2, Supplemental Content, http://links.lww.com/MD/K147 which list the DEGs between MUC16^MUT^ and MUC16^WT^ in LUAD patients). We conducted a univariate Cox regression analysis, revealing that 100 of 497 DEGs were considerably associated with the OS of patients (see Table S3, Supplemental Content, http://links.lww.com/MD/K148 which illustrates association between DEGs and OS of patients). Then, a risk score model was established to predict patient survival. The OS for patients in the high-risk group was significantly worse than in the low-risk group (Fig. 3A). Additionally, the predictive performance of IPM was assessed using time-dependent ROC curves. As shown in Figure 3B, the area AUC of the prognostic model for OS was 0.73 at 1 year, 0.77 at 3 years, and 0.80 at 5 years.

432 LUAD patients were enrolled in the meta-GEO LUAD cohort to determine whether the IPM was robust. Based on the same cutoffs as in the TCGA LUAD cohort, the meta-GEO HCC cohort patients were categorized as either high-risk or low-risk. The low-risk group had a significantly longer median OS than the high-risk group based on the results of the TCGA LUAD cohort (Fig. 3C). Furthermore, the IPM achieved an AUC of 0.80 at 1 year, 0.78 at 3 years, and 0.76 at 5 years (Fig. 3D).

### 3.3. Stratification analyses of OS for the IPM based on MUC16 status in the TCGA LUAD cohort

We performed a stratification analysis to determine whether the prognostic value of the IPM remains stable in different subgroups. The results indicated that the IPM was significantly involved in OS within the MUC16^WT^ and MUC16^MUT^ TCGA LUAD cohorts (Fig. 3E–G). Additionally, the correlation analyses revealed a negative association between the IPM risk score and survival time in both the MUC16^WT^ (r = −0.22, *P* = 3.1e-4) and MUC16^MUT^ subgroups (r = −0.2, *P* = 7.0e-3) (Fig. 3H). Furthermore, the univariate regression analysis suggested that the MUC16 mutation (HR = 1.05, 95% CI: 0.72–1.39, *P* = .039), the IPM risk score (HR = 3.88, 95% CI: 2.93–4.83, *P* < .001) and the Stage (HR = 1.68, 95% CI: 1.42–1.93, *P* < .001) had a significant association with OS. Moreover, the IPM risk score remained an important factor that affected prognosis in the multivariate Cox regression analysis. However, the MUC16 mutation did not remain statistically significant. Therefore, the IPM risk score could be an independent prognostic factor for LUAD patients (Fig. 3I).

### 3.4. Tumor microenvironment landscape within the low- and high-risk LUAD patients

**Figure 4.**
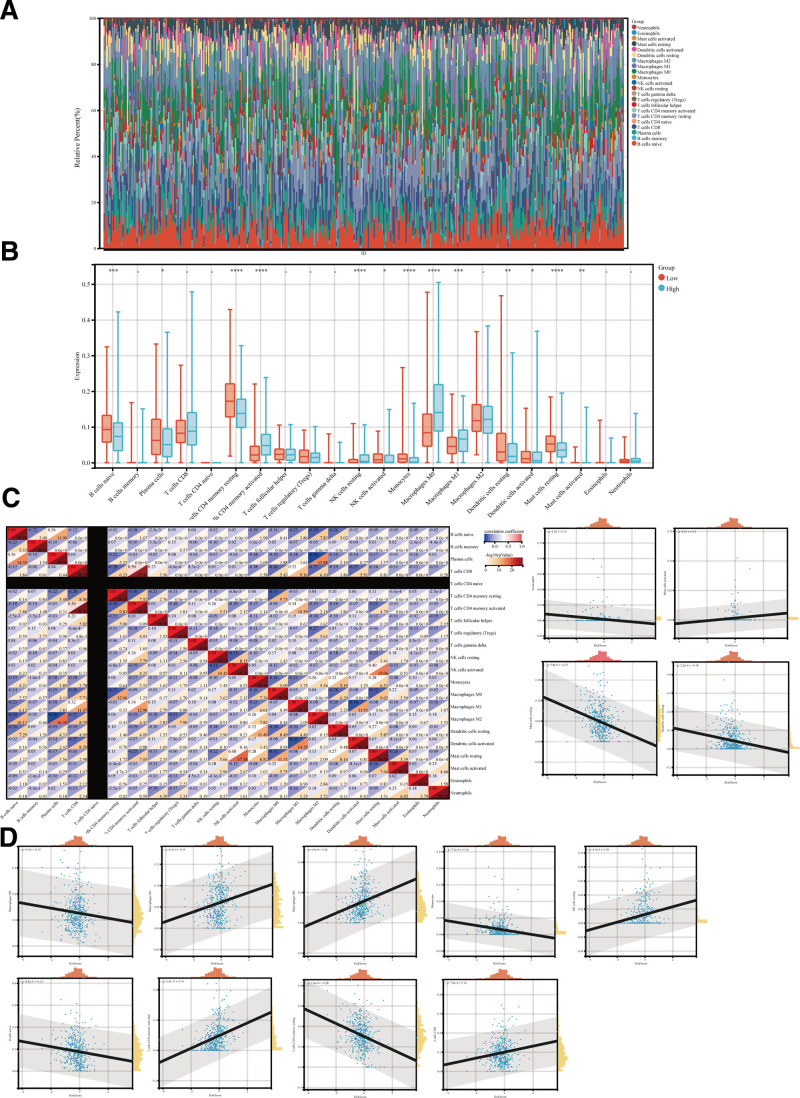
The landscape of immune infiltration within high- and low-risk lung adenocarcinoma (LUAD) patients. (A) The heatmaps summarize the relative proportion of immune infiltration among high- and low-risk patients. (B) Box plots represent differential immune cell expression between high- and low-risk patients. (C) The correlation matrix of 22 types of immune cell proportions. The colors red and blue depict positive and negative correlations, respectively. (D) The analysis of the correlation between risk score and immune cell infiltration.

Next, the CIBERSORT method was utilized with the LM22 signature matrix to determine the differences in immune infiltration between low- and high-risk LUAD cases. Figure 4A describes the results obtained from the 443 LUAD patients in the TCGA. The correlation matrix of immune cell proportions is depicted in Figure 4C. Figure 4B illustrates that low-risk LUAD patients had significantly higher proportions of naïve B cells, Plasma cells, resting memory CD4 T cells, activated NK cells, Monocytes, resting Dendritic cells, activated Dendritic cells, and resting Mast cells. Besides, the high-risk group cases had significantly higher infiltration of the activated memory CD4 T cells, resting NK cells, M0 Macrophages, M1 Macrophages, and activated Mast cells. Moreover, Figure 4D shows the correlation analysis between the risk score and different immune cells. We observed a strong correlation between the majority of the immune cells. For example, the M2 macrophages were negatively associated with plasma cells (r = −0.38). The activated dendritic cells were negatively related to M1 macrophages (r = −0.36), resting memory CD4 T cells were adversely associated with the M0 macrophages (r = −0.33), and M0 macrophages were negatively correlated with the resting mast cells (r = −0.32). Conversely, naïve B cells positively correlated with plasma cells (*R* = 0.36), and resting mast cells were positively associated with the activated NK cells (*R* = 0.40).

**Figure 5.**
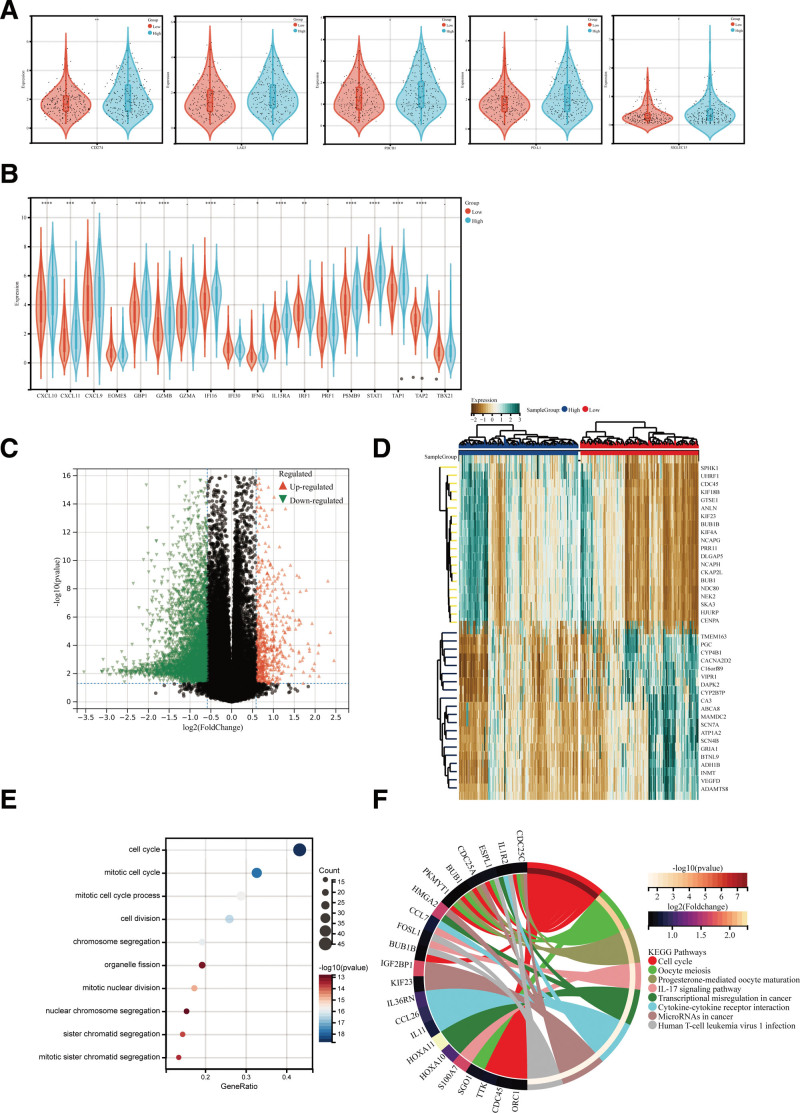
Enrichment analysis of the immune prognostic model. The violin plots help visualize significantly different immune checkpoints (A) and T-effector and interferon-gamma gene signatures (B) between the high-risk and low-risk patients. (C) The volcano plot represents the distribution of differentially expressed genes (DEGs) quantified. (D) The heatmap indicates the DEG expression with the threshold of |log2 Fold change| > 1, FDR < 0.05 and *P* < .05 in the cancer genome atlas (TCGA) cohort between the high-risk and low-risk patients. (E) The dots plot represents the GO signaling pathway enrichment analysis. The dots represent genes, and the size of each dot reflects the significance of gene expression changes. (F) The circular plot represents the Kyoto encyclopedia of genes and genomes (KEGG) signaling pathway enrichment analysis.

The immune checkpoint has a crucial role in tumor immune surveillance. Blocking the immune cells is an effective strategy with unprecedented results in lung cancer. Therefore, the relationship between prognosis risk score and immune checkpoint expression was analyzed. It revealed that the expression of PD-L1, LAG3, PDCD1, and SIGLEC15 was significantly upregulated in the high-risk LUAD group (*P* < .05) than in the low-risk LUAD group (Fig. 5A). We further researched whether IPM was involved in the T-effector and interferon-γ gene signature, which has an essential effect on activated T cells, cytolytic immune activity, and interferon-γ expression. Most T-effector and interferon-γ gene signatures demonstrated higher expression levels in high-risk LUAD groups (Fig. 5B). Thus, immunosuppressive microenvironments could be responsible for the poor prognosis of high-risk patients.

### 3.5. The different pathways in high- and low-risk group patients

GO analysis was performed to gain a deeper understanding of the biological effects of IPM. Limma (version 3.40.6) was used to differentially identify expressed immune genes between the low-risk and high-risk groups. Here, 465 dysregulated genes were found (|log2 Fold change| > 1, *P* < .05 and FDR < 0.05, including 124 upregulated and 341 downregulated genes within the high-risk groups (Fig. 5C and D) (see Table S4, Supplemental Content, http://links.lww.com/MD/K149 which shows immune-associated DEGs between the low-risk and high-risk group). GO and KEGG analyses could investigate the potential biological functions of these DEGs (Fig. 5E and F) (see Tables S5, http://links.lww.com/MD/K150 and S6, http://links.lww.com/MD/K151, Supplemental Content, GO and KEGG analysis results). These upregulated genes were predominantly enriched in the cell cycle, oocyte meiosis, progesterone-mediated oocyte maturation, IL-17 signaling pathway, transcriptional misregulation in cancer, cytokine-cytokine receptor interaction, cancer microRNAs, and human T-cell leukemia virus 1 infection pathway. They were also enriched in the biological processes mainly involved in the cell cycle, cell division, chromosome segregation, the mitotic cell cycle process, mitotic nuclear division, sister chromatid segregation, and mitotic sister chromatid segregation.

### 3.6. Predicting LUAD prognosis using an IPM-based nomogram

**Figure 6.**
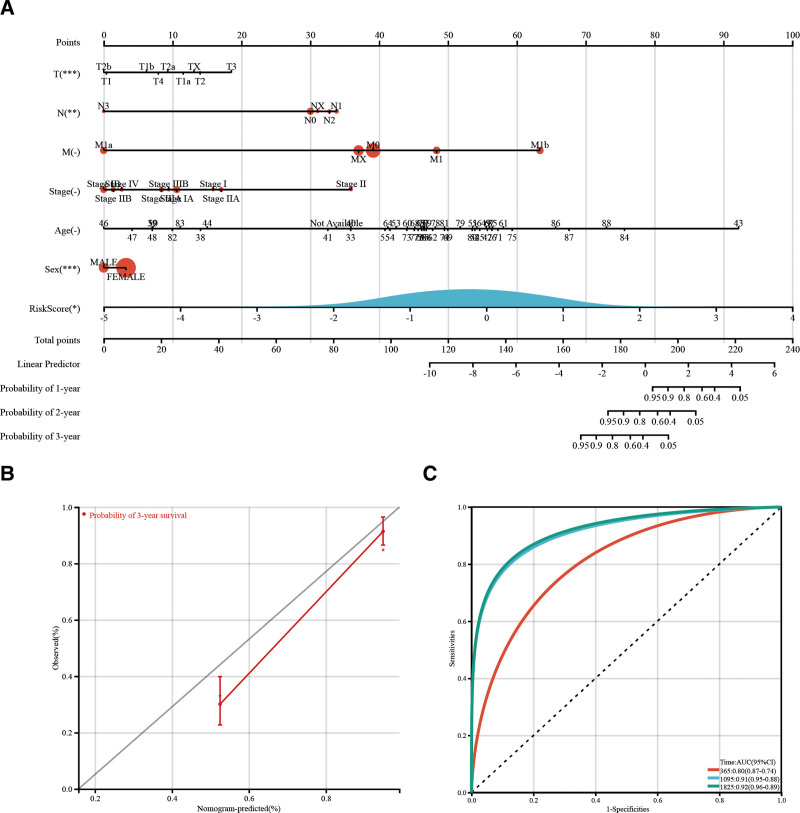
The association between immune prognostic model (IPM) and other clinicopathologic variables of lung adenocarcinoma (LUAD) patients within the cancer genome atlas (TCGA) cohort. (A) The nomogram for predicting the proportion of patients with overall survival (OS). (B) The nomogram calibration plots for the OS probability at 3 yr. The dashed line of 45° depicts the perfect nomogram prediction. (C) Time-dependent receiver operating characteristic (ROC) curve analyses for the immune prognostic model.

A nomogram was developed to incorporate the IPM and independent clinicopathological prognostic factors, such as age, sex, tumor grade, and TNM stage, and provide clinicians with a quantitative method for predicting prognoses of LUAD patients (Fig. 6A). The 7 variables were assigned points using a point scale based on the multivariable model. A straight line was drawn upward for each variable to determine the points. Then, the sum of the points was rescaled from 0 to 100. A straight line was drawn from the total point axis, and points assigned to each variable were added to predict the survival probabilities for 1 year, 3 years, and 5 years. Nomogram C-indices was 0.83 with 1000 bootstrap replicates (95% CI: 0.79–0.86). Depending on the calibration plots, the 3-year survival probabilities of the nomogram indicated a high degree of coincidence with the standard curves. Thus, there was a good agreement between the actual observations and the nomogram predictions (Fig. 6B). Additionally, ROC curve analyses revealed that AUC values for the 1-, 3- and 5-year survival rates were 0.80, 0.91, and 0.92, respectively (Fig. 6C). Therefore, the nomogram could guide the therapeutic strategy decision in the treatment and long-term prognosis observation of LUAD patients.

## 4. Discussion

Immunotherapy aimed at treating tumors involves provoking an antitumor-immune response. This includes invoking the immune system to inhibit tumor development, occurrence, and recurrence, with the characteristics of long-lasting and promising results with very few adverse effects.^[28]^ The tumor microenvironment has depicted tumor-associated neoantigens derived from non-synonymous somatic mutations as the primary targets of cytotoxic T lymphocytes.^[29]^ Lei Zhang et al observed that mutations in MUC16 are associated with better ICI response and outcomes in solid tumors.^[30]^ Furthermore, the MUC16 mutation is associated with better prognosis, and higher TMB in glioma and hepatocellular carcinoma patients have been verified. However, there is no systematic exploration of the potential role of MUC16 mutation in LUAD therapy.

In our study, the somatic mutation landscapes of LUAD were demonstrated in 516 samples from the TCGA cohort. Afterward, 20 genes were frequently mutated in the 2 databases. MUC16 ranked the third-highest mutation frequency after TP53 and TTN. TMB can be used as a biomarker in small-cell lung cancer to monitor immunotherapy effectiveness. High TMB tumors possess higher levels of neoantigens, which the immune system recognizes as antigens.^[7]^ TMB was remarkably improved in the MUC16 mutation group. Next, we divided the LUAD patients into MUT and WT groups to identify the effect of MUC16 gene mutations on tumorigenesis in LUAD. The immune signaling and cancer pathways were enriched in the MUC16^WT^ group due to the GSEA analysis of LUAD samples with and without MUC16 mutations. Differential expression analysis revealed 335 downregulated and 162 upregulated DEGs in MUC16^MUT^ compared to MUC16^WT^ LUADs. Besides, a negative association was observed between IPM risk score and survival time in the MUC16^WT^ and MUC16^MUT^ subgroups. Multivariate analysis indicated that the IPM was an independent prognostic factor after modifying clinical characteristics. Area under the curve of the ROC curve revealed that the risk signature was satisfactory, indicating its predictive capability.

The immune system plays a vital role in the occurrence, development, and prognosis of most tumors while forming a specific tumor immune microenvironment. Tumors evade detection and destruction by manipulating the immune system. They also downregulate costimulatory molecules on tumor cells, increase the expression of immunosuppressive molecules, and dysregulate T cells and APCs.^[31]^ This study analyzed the immune mechanisms between the low- and high-risk group patients, and cancer immunotherapy aims to improve antitumor immune responses. The results indicated that the proposed approach was effective and efficient. High-risk LUAD patients had higher fractions of CD8 T cells, activated memory CD4 T cells, resting NK cells, M0 Macrophages, M1 Macrophages, and activated Mast cells (*P* < .05). Additionally, the correlation matrix results indicated that plasma cells were positively associated with naïve B cells and negatively with M2 macrophages. Moreover, M0 macrophages were negatively correlated with resting mast cells and memory CD4 T cells. Furthermore, the immune checkpoint expression between the low- and high-risk groups was investigated. Patients with high-risk scores had increased levels of immunosuppressive molecules, PD-L1, LAG3, PDCD1, and SIGLEC15. NSCLC tumors expressing PD-L1 have been used as anti-PD-L1/PD-1 immunotherapy biomarkers. Thus, PD-L1-positive NSCLC is associated with enhanced response to anti-PD-L1/PD-1 immunotherapy.^[32]^ Meanwhile, the high-risk LUAD patients showed significantly higher expression of the T-effector and interferon-γ gene signature than the low-risk patients (*P* < .05). The results showed that immune dysregulation could induce the OS differences between patient subgroups stratified by the IPM. Therefore, a poor prognosis for high-risk patients could be related to the stronger immunosuppressive microenvironment and immune checkpoint expression than in low-risk patients. LUAD is more likely to grow and progress due to these differences, causing poor prognosis.

In addition, the differentially expressed immune genes between high-risk and low-risk groups were analyzed with GO enrichment and KEGG pathway analyses. These DEGs are enriched in many immune systems, such as the hematopoietic cell lineage IL-17 signaling pathway, Fc epsilon RI signaling pathway, and the intestinal immune network for IgA production, complement, and coagulation cascades. A nomogram can develop a statistical model that predicts an intuitionistic and accurate scoring system to estimate the OS of LUAD using IPM and independent clinicopathological prognostic factors. The nomogram also established the prognostic value of IPM in LUAD.

Further research must be conducted to address the limitations of our study. Initially, we could not determine whether MUC16 mutation was also associated with the prognosis and tumor immunity in Chinese patients and whether it could lead to the same immune response due to the lack of clinical data in the ICGC database. Thus, data from publicly accessible databases were utilized to conduct these informatics analyses, and further experimental validations are needed. Moreover, the characteristics of each tumor are unique, leading to inter-patient heterogeneity as well as intra-patient, which could result in sampling bias.

Moreover, in this study we simply discussed the potential of MUC16 as a new therapeutic target and did not perform a thorough analysis of functional enrichment or loss in the MUC16 mutation. Second, while we evaluated the prognosis of the MUC16-MUT and MUC16-WT groups, the prognostic analysis of the MUC16-MUT group could not distinguish between different therapies. However, varying treatments may have different outcomes, and the different TP53 mutation types of LUAD have a variable prognosis. Finally, our study has not been confirmed in further experiments. Nevertheless, our study provides valuable information and insights for future LUAD research.

## 5. Conclusion

MUC16 mutation was related to higher TMB, better patient prognosis, and significantly improved immunotherapy prognoses. It is the first study to describe the association of IPM with MUC16 mutations, which can be used as a guide. IPM also provides an immunological perspective to elucidate the mechanisms determining the clinical outcome in LUAD. In summary, our study demonstrated the potential immunotherapeutic and prognostic value of MUC16.

## Author contributions

**Funding acquisition:** Hao Chen.

**Methodology:** Jing Liu, Tingya Liu.

**Project administration:** Quangang Chen, Ankang Hu.

**Writing – original draft:** Tingjun Liu.

**Writing – review & editing:** Lianlian Wu, Bao Zhu, Dandan Qiao, Yuhua Zhu.

## Supplementary Material
